# Supplementary material for: Patterns of Intron Gain and Loss in Fungi
Source: PLoS Biol. 2004 Nov 30;2(12):e422. doi: 10.1371/journal.pbio.0020422 (PMC532390; doi:10.1371/journal.pbio.0020422)
Supplement: Table S1 — Also available at http://genes.mit.edu/NielsenEtAl/. (4.3 MB ZIP). [file pbio.0020422.st001.zip › NielsenEtAl/html/35.html]

AN4385.1.NCU01335.1.MG04100.1.FG10381.1


```
 CLUSTAL W (1.82) Multiple Sequence Alignments - Introns Inserted


Sequence 1: NCU01335.1	1505 aa
Sequence 2: FG10381.1	1396 aa
Sequence 3: MG04100.1	1484 aa
Sequence 4: AN4385.1	1322 aa
Alignment Length: 1595 aa
Number Identitical Residues: 575 aa
Alignment Score (without introns) 28395


MG04100.1 	MTPPGG---FAERAYSGSNGKAVASSSVSNSGGSRTPGTPRKNSTANLAREKALQDPGLR
NCU01335.1	MAPNPPGSFSTERSYHGGNGKAAAVVATK------VPGTPRREAGG-HGREKAVQDPGLK
FG10381.1 	MVP--------ERQYHGNGRPAIASRERS------VPGTPSRKEK--IRESSALQDPGLK
AN4385.1  	MVS---------RSNEGPEAPHPAS---------RTPGAPAKGRLTRLGSSPSKRDDKAK
          	*..         *   *      *           .**:* :        . : :*   :

MG04100.1 	DY0R---------~-LGDCIGKGAFGSVYKAFNW~GTGEAVAVKQIKLVDVPRSELRMIE
NCU01335.1	DY0R---------~-LGDCIGKGAFGSVYKAFNW~GTGEAVAVKQIKLVDVPKSELRMIE
FG10381.1 	DY~VRIPLSPMLL0RLGECLGKGAFGSVYKAFNW~GNGEAVAVKQIKLADLPK-------
AN4385.1  	DD~R---------~-----MGKTSAKDVAELKDY0----------IKLADLPKSELRVIM
          	*                  :** :  .* :  ::           ***.*:*::.     

MG04100.1 	0AEIDLL~KNLH0HDNIVKYIGFV~KTSDCLNIILE2YCENGSLHSICKAYGKFPENLVG
NCU01335.1	0AEIDLL~KNLH0Y------IGFV~KTADCLNIILE2YCENGSLHSICKAYGKFPENLVG
FG10381.1 	~-----M1KSIF~S------KTFT2YETDES----R~YCENGSLHSICKSYGKFPENLVG
AN4385.1  	0LEIDLL~KNLD0HPNIVKYQGFV~KSAETLNIILE2YCENGSLHSIAKNFGRFPETLVG
          	  . . : *.:    .  .   *.   ::  .   . **********.* :*:***.***

MG04100.1 	VYMTQVLQGLQYLHDQGVIHRDIKGANILTTKDGKVKLADFGVSTSTLAG-PDKEAQVVG
NCU01335.1	VYMTQVLQGLQYLHDQGVIHRDIKGANILTTKDGTVKLADFGVSTSTLAG-PDKEAQVVG
FG10381.1 	VYMTQVLQGLQYLHEQGVIHRDIKGANILTTKDGTVKLADFGVSTSTLAGGQDKEAQVVG
AN4385.1  	VYMSQVLHGLLYLHDQGVIHRDIKGANILTTKEGLVKLADFGVASRTTGL---SESSVVG
          	***:***:** ***:*****************:* ********:: * .    .*:.***

MG04100.1 	TPYWMAPEIIQLSGATSASDIWSVGCTVIELLQGKPPYHNLAPMPALFAIVNDDHPPLPE
NCU01335.1	TPYWMAPEIIQLSGATSASDIWSVGCTVIELLQGKPPYHHLAAMPALFAIVNDDHPPLPE
FG10381.1 	TPYWMAPEIIQLSGASSASDIWSVGCTVIELLQGKPPYHNLAAMPALFAIVNDDHPPLPE
AN4385.1  	TPYWMAPEVIELSGATTASDIWSLGCTVIELLEGKPPYYNLQPMPALFRIVNDDHPPLPQ
          	********:*:****::******:********:*****::* .***** **********:

MG04100.1 	GVSP0AARDFLMQCFQKDPNLRVSAKKLQRHPWIVGSRRSDAPVSKAPSNFSEAVEEVKQ
NCU01335.1	GVSP0AARDFLMQCFQKDPNLRVSAKKLLRHSWIQGCRRSDAPISKAPSNFIQAVEEVKQ
FG10381.1 	GISA0AARDFLMQCFQKDPNLRVSARKLLRHAWITGCRRTEAPVSRKPANFSDAVEEVKQ
AN4385.1  	GASP0AVKDFLMQCFQKDPNLRVSARKLLKHPWIVNARRSDSVVPKKSTEYEEAVKSVQE
          	* *. *.:*****************:** :*.** ..**::: :.: .::: :**:.*::

MG04100.1 	WNKALKSSEH-GLRVSIGPDGAHHSGSNGTKGTTVDGHRLNVITNIKGPLTLAKPRPMAD
NCU01335.1	WNKALKSSEA-TPRTSIGSDHGNPSTQN--RNASGQ-HRPNLSISTKVPPQQAKPKANAE
FG10381.1 	WNKALKSSEP-NLRASLGSETG-PSTRL--QGGT-----------------PAKGSLSLA
AN4385.1  	WNEALRSPESNALRRGTRNENQNPPSLRLDTRHTPT----------KVTLPSPVSRIVAD
          	**:**:*.* .  * .   :    .        :            . .   .       

MG04100.1 	AFRSPEIPD1N~D-NWDNDFATAISPSALHLPHLKPQDNFGGLLSSDRLKAFASID----
NCU01335.1	VFRSPAVEA~D1DDNWDNDFATAISPSALHLPHIKGQDNFGGLLSSDRLKAFASID----
FG10381.1 	KLRSGAGAF~N1DDNWDDDFATSISPSALHLPHIKPQDNFGGLLSSDRLKAFASIN----
AN4385.1  	KFSSPSSGE~E~D-NWDDDFATAISPSALQLPHLRPHDNFGGMLSSEKLKAFASLDGTVL
          	 : *      : * ***:****:******:***:: :*****:***::******::.:  

MG04100.1 	DSRNLSADWDDDLGGSEQLVTIKAPRAQLSDPDMQQTIRPTPRKNDHSEKREKKASLPDF
NCU01335.1	SSREDTENWDEDFGG-EF----------------EQTIRPLPKRSDR--VSDSKSSQQGH
FG10381.1 	DGRNESNTYDDDFEG-ELMTIKGLGQWQDSSDLQEQTIRPTPRKPTK--APEP---VKVH
AN4385.1  	KSDESFEESDDPFKGSLLAG----------EHDPLKTIRPPPSQQANSGTSQSQNGPYGA
          	.. :     *: : *:              .    :**** * :  .:   : . .    

MG04100.1 	KLQQPNKTKQSRTS-AKSPTKPHFPAKFTIPPRPESMFREHSVEDYSDILEDNDHVFAEK
NCU01335.1	RRQRSSKTTISSASQTKSPVRTQFGNKFELPPRPESIYREQSTEDYSDLFGDTDSVFSSR
FG10381.1 	SRNKSSTSKAVGGGRTKSPTKSHLNNKFELPLRPDLAYREQSVDDFSDLFVDNDNVFTHN
AN4385.1  	HMRRGPPLNTAITPVYGGQMLQNSSSPIRQQ-RPPLFYKENSVEDYSDLISANEDVLDRK
          	  .:    .        .    :    :    **   ::*:*.:*:**::  .: *:  .

MG04100.1 	MGLVP~K~-~-----DSPQLFHPSDLTSLPRSMQ---PPNGGSVRRAAQPRPSVLPDRNM
NCU01335.1	LNVAV~K0-~-----DAPQLFHPSDLTSLPRSTASPVNGSSGRRQSASARSPSAPEKPAV
FG10381.1 	VNQAV~R~R0NSRQSDAPQLFHPSDLTSLPRSMQ---ESSSGSIKKKPLSRPSVLPDAPM
AN4385.1  	ISAFQ0E~-~---------SDEHKDLADTGRSRE----VIRYQSSYEQEDTPRIGRQISV
          	:.    .              . .**:.  **                   *    .  :

MG04100.1 	RRTRSSIEIQRFAENESDEDFSDIFGPSD-TLTERDESDRGSEDGGTGGGRMILSKLSNN
NCU01335.1	RRTRMTVEITKFAEDEGDEDFSDIFGAEPESFTKPPKSDEGSEDGH---QLMLLSKLSNN
FG10381.1 	RRTRSGIEIQKFAEDD-DEDFSDVFGPES-SIAEKEESEKGSEDG----GLMLMSRVSSS
AN4385.1  	KRYRGTVEIQQFAENEHDEDFSDILGVDG-VTLDKAESDDGSNKS----TLMLNTKLSNN
          	:* *  :** :***:: ******::* .     .  :*: **:..      *: :::*..

MG04100.1 	SWLGDDEDEDDPFALLDPGWDEMDLEANIARDRHARLSHKVEELVRSLKTTEGEDTLLVV
NCU01335.1	SWLGDDEDEDDPFAMMDPGWDEMDLEANIARDRHARLAEKVEERVKSLKPTESEDILADL
FG10381.1 	SWLGDEEDEDDPFAYMDPGWDEMDLEANIARDRHARLAERVEDLVRSLKTTEGDDALAEF
AN4385.1  	SWLGDLDDEDDPFALLEEGLDETDLEANIARDKHARLRSQVEGLVGSLKTSQDEEVLGDI
          	***** :******* :: * ** *********:****  :**  * ***.::.:: *  .

MG04100.1 	SEEL0LSLLWENGDVKDLIISAHGLLPILEILEPCTVKSRQHMILQLLKIVNA0IILDDV
NCU01335.1	AEDL~LNLLWENSEVKDLIISAHGLLPILEILEPCTVKSRQHMILQLLKIVNA0IILDDV
FG10381.1 	SEDL0LALLWENSEVKNLIISAHGLLPILEILEPCTVKSRQYMILQLLKVVNA0IILDDV
AN4385.1  	SEQL~LAVFCDFPETKNIIISAHGMLPILEILDLCR---RRDITLCLLKIVNA~IIYDDY
          	:*:* * :: :  :.*::******:*******: *    *: : * ***:*** ** ** 

MG04100.1 	ELQENLCFVGGIPIITKFAARQYSNEIRLEAAAFVRQMYQTSTLTLQMFVSAGGLNVLVE
NCU01335.1	ELQENLCFVGGIPIITKFAARQYSNEIRLEAAAFVRQMYQTSTLTLQMFVSAGGLNVLVE
FG10381.1 	EIQENLCFVGGIPIITKFAARQYSDEIRLEAAAFVRQMYQTSTLTLQMFVSAGGLNVLVE
AN4385.1  	EIQENLCFVGGIPIINEFAAKKYPREIRLEAAAFVQQMYQTSTLTLQMFVSAGGLNVLVE
          	*:*************.:***::*. **********:************************

MG04100.1 	FLDEDYDASRDLVLIGVNGIWNVFELQ~GPTPKNDFCRIFSRSKILDPLALVLHKVLDED
NCU01335.1	FLDEDYDMSPDLVLIGVNGIWNVFELQ0GPTPKNDFCRIFSRSKILDPLAAVLHKVLDED
FG10381.1 	FLDEDYDNTRDLVLIGVNGIWNVFELQ~GPTPKNDFCRIFSRSKILYPLALVLHRVVDDD
AN4385.1  	FLEDDYEDERDLVLVGVNGIWSVFELQ0GSTPKNDFCRILSRSSVLDPLSLVLSRVLDEE
          	**::**:   ****:******.***** *.*********:***.:* **: ** :*:*::

MG04100.1 	DKSELTELVEGRIVNIFYLFSQAENYVKEVVAERQVLKT1------VLRDLRRMSPIHQI
NCU01335.1	DRDELSELIEARIVNIFYLFSQAENYVKEVVAERFVLKT1------VLKDLRRMTPAHQI
FG10381.1 	DEDELGELVEGRIVNIFYLFSQAENYVKEVVADRQVLKS~------VLRDLRLMTPIHQI
AN4385.1  	--GELAEVVEGRIANIFFIFSQAENHVKEMVSERTVLHS1TDDGLGVLKELKRMTPAHQI
          	  .** *::*.**.***::******:***:*::* **:: :... .**::*: *:* ***

MG04100.1 	TMLKFIKNLSMLSLTLESLHSADAIDFLIELLSYSMKKGHKHFREISNQVLNTMFNLCRL
NCU01335.1	TMLKFIKNLSMLSTTLESLHSADAIDYLIDVLSNSMKRGQQHFREISNQVLNTMFNLCRL
FG10381.1 	TMLKFIKNLSMLSTTIESLHSADAIESLINLLGYSMRKGQTHFREISNQVLNTLFNLCRL
AN4385.1  	TMLKFIKNLSMLSTTLDSLQNSNAIDVLTDLLRSTIKR--PHFREVSNQILNTIYNMCRL
          	************* *::**:.::**: * ::*  ::::   ****:***:***::*:***

MG04100.1 	SKERQEYAAVNGIIPLLLKIMQTDRPPKEFALPILCDMAHSGSKGRRYLWQNKGLDFYVS
NCU01335.1	SKERQEYAASNGIIPLLLKIMKTDRPPKEFVLPILCDMAHSGSKGRRYLWQNNGLEFYVS
FG10381.1 	SKERQEDAAVHGIIPILLKIMQTDRPPKEFALPILCDMAHSGSKGRRFLWQNKGLNFYVS
AN4385.1  	NKSRQEDAALNGIVPLLQKIVKTERPLKEFALPILCDMAHSGKVGRRELWRNKGLAFYIS
          	.*.*** ** :**:*:* **::*:** ***.***********. *** **:*:** **:*

MG04100.1 	LIADQYWQVSALDSISVW~LQEETAKVETHLLDG-----KFTAAITSSFNSIKTNAFDSG
NCU01335.1	LLADQYWQVTALDAIFVW~LEKETAKVEHHLLDG-----NFTTAITSCFNTNKANAFDAN
FG10381.1 	LLTDQYWQVTALDAILVW~LQEETANVESHLADG-----GFTRAINSCFNTNRVNAFDSN
AN4385.1  	LLSDPYWQVTALDAIFIW2LQEETAKVEEHLLENRYDQPSFTDAIVRCLTLSKANAFEN-
          	*::* ****:***:* :* *::***:** ** :.  ... ** **  .:.  :.***:  

MG04100.1 	VLEPLIKILRLSPSLAASLAKSEMYSGLAQKLGHKKAQVRLNLLRLVRNIMDACVSE-GG
NCU01335.1	LLEPLLKLVRLSPSVAASLAKVEMYAGIAQKLSHKKAVVRLNLLRLVRSIMDGCEVNNTP
FG10381.1 	LLEPLLKLLRLSPSVAASLAKPEMFAGIAQRLGHKKAVVRLNLLRLVRTIMDACEPG-LG
AN4385.1  	ILEPLQKLLRLSPPIASTLARPDLFSRLGQKLHHSKAAVRLNLLRIISSICDSSEQQ---
          	:**** *::****.:*::**: :::: :.*:* *.** *******:: .* *..      

MG04100.1 	GSSMSTASSGRQLRALFEQIRVLADKDPAVLVRNLASELLRSHLDPDGVTNPIGLTTSTS
NCU01335.1	MATLATSTAGRALRVLFDDIQILADKDPAVLVRNLASEIVRSHIDID-------LQHDVA
FG10381.1 	NGDGTRSLNSSQVRSLMATIQILAEKDTAVLVRNLASELVRSNIDTT-------VRPSEG
AN4385.1  	-GGLLASYG------LLDSIRELEH-DPAILVRDMAGKLIQSSERND-------------
          	 .    :        *:  *: * . *.*:***::*.::::*                  

MG04100.1 	AVGN-GANPLSSAVP-PSSSGAPSSRSRSGPRRTSSYTPPGLSSSASAPPQTPTHHQRPS
NCU01335.1	ALGALNMGAGPSAIPNPLNNLSSGPRSRSGPRRNTSYTPPGLHASMSM-PPTPTHGHRAS
FG10381.1 	AA---------TAVP-------SSSRQR-GSRRNNSYTPPSLQTSVSAGPQTPTHRTRPS
AN4385.1  	-------------------SYGLCKLKPNVRRGSTSATSPGLLANQSAPVTPQLSRQNQS
          	                   . .    . .  * ..* *.*.* :. *    .     . *

MG04100.1 	QSSSIYTEVAVTATPRRSAAIAQERDSGGVFRPRSRDGPGSSGSSSSIPRRVSGEGGA--
NCU01335.1	-HSSSAAYIEVASTPKRSGISLAQER-EAMYRPHSRDGGVP---LINIPRRVSQDTSAGI
FG10381.1 	-LAN-NAYVEVAASPRRSAAVERERE-GMIYRPRSKDG------PTGIPRRISGEFAP--
AN4385.1  	-----KGYYDSRETQRRPRSAISGSA--LALRPGSRDG--------PTPGIVGGANGS--
          	             : :*.             ** *:**          *  :.   ..  

MG04100.1 	------------------ILLGGKNGVSSRLPR----------------------TSSAY
NCU01335.1	PSLSSSMGSAGGLGTPSRMRLPRMSMTQYSSPRPSLSSAATAPGAVSATGMGIGNSSIAS
FG10381.1 	----------------VKSRLPRTSLVTSSSSRAGVG--------ISAN----GNSPALS
AN4385.1  	---------------------------------------------------------AGA
          	                                                            

MG04100.1 	GRQSISMAQGRSESSSSNKENTNITQMG-------------GGSDSRYHTPTAANFDQ--
NCU01335.1	SRAERERERSESSLSNHNKENYGRIRSGSSVAGPEARILGGGGLYSVSPTGSAVSLSSSF
FG10381.1 	SRHDITMARSDSSLS--NKENVGRAP---------------GSRDGIGSRDSSSSLAS--
AN4385.1  	SRNRIARGVSNRLSHIELLPND-------------------DDRIPSSITRRSSVLPR--
          	.*       .          *                    ..         :  :    

MG04100.1 	--------HDLDWE-RERDRQRGDRAERVAAQQHKRRSRMTASSSDIRHGAPSR
NCU01335.1	QGSGGGGSEGFSRSYGARESSFSVMGGSAGAGNAKRRSRAPSSDMRWS------
FG10381.1 	--------PGLS---GVPERAGIIS---------KRRSRMP-TEPKWP------
AN4385.1  	------------------------------------RRRLTQFEAERGS-----
          	                                    * * .  .
```
